# Acute Right Ventricular Failure in the Setting of Acute Pulmonary Embolism or Chronic Pulmonary Hypertension: A Detailed Review of the Pathophysiology, Diagnosis, and Management

**DOI:** 10.2174/157340308783565384

**Published:** 2008-02

**Authors:** Jennifer Cowger Matthews, Vallerie McLaughlin

**Affiliations:** Department of Internal Medicine, Division of Cardiology, Section of Pulmonary Hypertension, University of Michigan Health Systems, Ann Arbor, Michigan, USA

**Keywords:** Right ventricular failure, shock, pulmonary hypertension, pulmonary embolism.

## Abstract

The right ventricle (RV) is integral to normal cardiac function, but receives little attention in the medical literature. The etiologic causes of acute RV failure often differ from those encountered in left ventricular dysfunction. Thus, RV failure frequently requires diagnostic procedures and management strategies that differ from those routinely used in the management of intrinsic left ventricular dysfunction. In this summary, the structure and function of the RV will be reviewed, concentrating on the pathophysiologic mechanisms behind the development of RV dysfunction. We will then focus on two distinct populations of patients who are at risk for acute RV failure: those with chronic pulmonary arterial hypertension (PAH) and those with acute pulmonary embolism. In chronic PAH, we will examine clinical circumstances common to hospitalized patients that may provoke acute RV decompensation, as well as pharmacologic therapies that are unique to RV failure management in PAH. Individuals with acute RV failure in the setting of pulmonary embolism represent a group with particularly high mortality, and the specific diagnostic and management strategies that are important for improved survival will be discussed.

## INTRODUCTION

The right ventricle (RV) differs structurally and functionally from its better studied neighbor– the left ventricle– often with a different etiology for its functional impairment. Thus, the diagnosis and management of acute RV failure often requires tactics that are distinctly different from those employed in patients with isolated intrinsic left ventricular dysfunction. In this article, we will overview the etiologies and pathophysiologic mechanisms behind the development of RV failure. We will also discuss the diagnosis and management of acute RV failure in two important patient populations: hospitalized patients with pre-existing chronic pulmonary hypertension and those with *de novo* RV dysfunction secondary to acute pulmonary embolism.

## PATHOPHYSIOLOGY OF ACUTE RIGHT VENTRICULAR FAILURE

In 1936, Fineberg and Wiggers hypothesized that “circulatory failure following obstruction of the pulmonary circuit had no other cause than fatigue of the right ventricle [1].” Decades later, this postulation holds true. While the term pulmonary arterial hypertension (PAH) identifies a population of patients with various degrees of pulmonary arteriopathy and elevated vascular resistive indices, it is the function of the RV that determines much of the morbidity and mortality that occurs in PAH. Acute adverse disruptions in cardiac hemodynamics can provoke RV decompensation at a rate and threshold dependant on the structure and function of the underlying RV myocardium. Factors that affect RV afterload, RV preload, and left ventricular (LV) function directly or indirectly impact the performance of the RV myocardium.

### Influence of Afterload on Right Ventricular Function

**Fig. (1):**
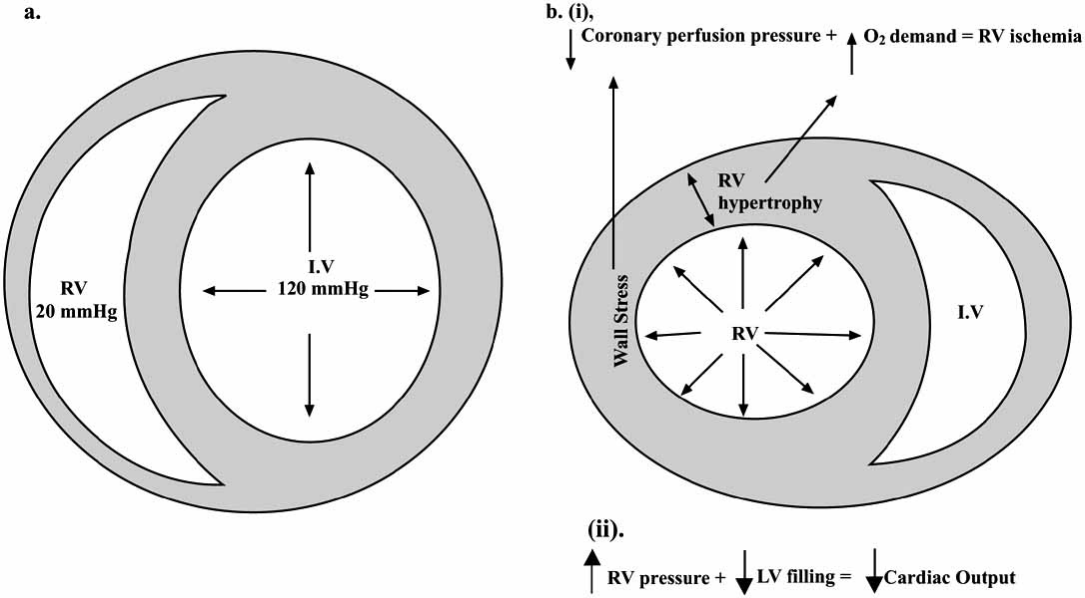
Short axis view of the right (RV) and left (LV) ventricles in a patient with normal (a) pulmonary pressures and in a patient with pulmonary hypertension (b). In normal individuals, note the crescent shape of the RV and the thin wall. In pulmonary hypertension, elevations in RV pressures increase myocardial wall stress. As the RV hypertrophies, myocardial oxygen demands also increase, culminating into a myocardial supply/demand mismatch (i). With increased RV filling pressure and volume, the septum is displaced towards the LV, reducing cardiac output (ii). Reproduced with permission from Chin *et al*. [4].

In normal individuals, the pulmonary circulation is a low resistance, high capacitance system capable of accommodating a three- to four-fold increase in RV stroke volume without significantly raising pulmonary pressures. While the LV is mostly composed of fibers arranged in parallel creating a concentric-shaped ventricular cavity capable of withstanding high afterloads, the RV is crescent-shaped (Fig. **1a**) with most of its fibers arranged in series [2]. The RV free wall is thinner (<0.6 cm) and has a much lower volume-to-surface area ratio than the LV, allowing for increased RV compliance.

**Fig. (2):**
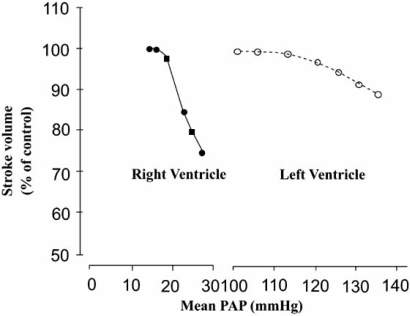
Impact on right (RV) and left (LV) ventricular stoke volumes with increased afterload. Acute increases in RV afterload lead to substantial decreases in RV stroke volume compared with that of the LV. PAP= pulmonary artery pressure. Reproduced with permission from Chin *et al*. [4].

However, the crescent-shaped geometry of the RV also makes the chamber poorly tolerant of acute elevations in afterload. While chronic pulmonary hypertension stimulates adaptive changes in the RV myocardium that help maintain RV stroke volume, this does not occur in the acute setting. In normal individuals, the RV is unable to acutely generate a mean pressure >40 mmHg, and stroke volume decreases linearly as RV afterload increases (Fig. **2**). Rapid elevations in afterload can cause acute RV dilation, damaging the contractile sarcomere apparatus with resultant RV failure.

### Influence of Preload on RV Function

Right ventricular systolic function is also dependant on RV preload. Elevations in RV preload most commonly occur secondary to an elevation in intravascular volume or tricuspid regurgitation. With progressive RV dilation, the RV begins to function on the descending portion of the Frank-Starling curve, and a vicious cycle of “auto-aggravation” ensues [3]. As RV end-diastolic volumes and pressures increase, RV wall stress increases leading to reduced RV stroke volume (Fig. **1b**). Elevated RV end-diastolic volumes also promote tricuspid annular dilatation, which worsens tricuspid valve insufficiency and decreases cardiac output.

### Influence of Ventricular Interdependence on RV Function

Ventricular interdependence is the final factor that affects RV systolic function [3-5]. In order to generate a continuous, low pressure stroke volume during systole, the normal RV has three phases of systolic contraction: 1) contraction of the papillary muscles, 2) inward movement of the RV free wall towards the interventricular septum, and 3) contraction of the LV with subsequent medial displacement of the interventricular septum towards the RV [3]. Perturbations in one ventricle may directly impact the neighboring ventricle (Fig. **1b**) [3-4]. For example, increases in RV end-diastolic pressure and volume cause the interventricular septum to shift towards the LV. Even in the setting of normal LV contractility, the reduced LV cavity size reduces stroke volume. Elevations in RV pressures also increase RV wall stress. This, in addition to increased myocardial oxygen demand in the setting of RV hypertrophy, leads to a myocardial supply/demand mismatch.

In converse, increases in LV volumes in the setting of LV systolic dysfunction shift the interventricular septum towards the right ventricle, increasing RV end-diastolic pressures. This exacerbates tricuspid regurgitation and wall stress, which initiates the “auto-aggravation” cycle.

## ACUTE RIGHT VENTRICULAR FAILURE IN CHRONIC PULMONARY HYPERTENSION

Acute RV failure in the setting of chronic pulmonary hypertension occurs as a consequence of complications that develop secondary to PAH disease management, from an acute worsening of previously stable pulmonary hypertension, and/or from the development of new cardiac abnormalities that are either primary in etiology or secondary to preexisting PAH. In the following section, the etiologies precipitating acute RV failure in hospitalized chronic PAH patients will be discussed, along with the strategies that should be employed to diagnose RV failure. Finally, we will review indications for vasopressor and inotrope support in RV failure and highlight vasodilator therapies unique to PAH management.

### Etiologies of Acute Right Ventricular Failure

While many patients with chronic PAH may appear clinically compensated, some degree of underlying systolic and/or diastolic RV myocardial dysfunction exists. The tenuous nature of advanced chronic PAH allows RV failure to develop acutely with few insults. Successful management of acute RV failure is predicated upon rapid identification and management of its underlying potentiating causes.

#### Myocardial Ischemia

**Fig. (3):**
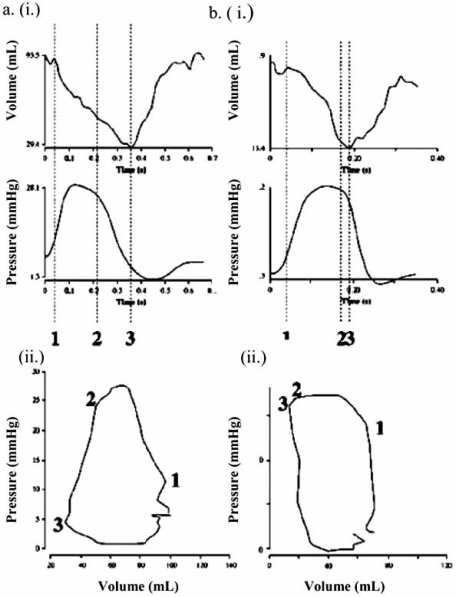
Pressure volume changes versus time during the contraction cycle (i) and the corresponding pressure-volume loop (ii) are shown for a normal patient (a) and a patient with elevated pulmonary afterload (b). In normals (a), the pressure-volume loop of the RV is more triangular than that of the LV with a poorly defined isovolumic contraction phase. In the presence of elevated RV afterload (b), the pressure-volume loop resembles that of the LV. Reproduced from Mebazza *et al.* [3] with permission.

Even in absence of significant obstructive atherosclerotic coronary disease, patients with chronic PAH are at risk for RV myocardial ischemia due imbalances in myocardial supply and demand that can develop with perturbations in RV preload and afterload, precipitating acute RV failure [6]. In normal individuals, the pressure-volume loop of the RV (Fig. **3a**) is more triangular than that of the LV (Fig. **3b**), with *short *isovolumic contraction and relaxation periods, allowing for sustained low pressure ejection [3, 7,8]. In this low pressure circuit, right coronary artery perfusion occurs during *both* diastole and systole [9]. In right ventricles subjected to chronically elevated afterloads, the pressure-volume curve begins to look like that of the LV with a *longer* isovolumic contraction interval and cessation of systolic ejection at the onset of a prolonged isovolumic relaxation period [7,8]. Increased RV afterload and a prolonged isovolumic contraction time result in an increase in myocardial oxygen demand and a reduction in right coronary artery systolic perfusion [9]. Elevated end-diastolic pressures in the setting of RV hypertrophy, intravascular volume overload, and tricuspid regurgitation reduce diastolic right coronary perfusion and increase wall stress. Individuals with chronic PAH poorly tolerate coronary lesions and can even develop RV ischemia in the absence of significant obstructive coronary disease. 

In chronic PAH patients with evidence of myocardial ischemia, measures should be undertaken to reduce myocardial oxygen demands and improve coronary blood flow. Hypoxemia should be improved with supplemental oxygen. In patients with volume overload, diuresis should be attempted to reduce RV wall stress and hepatic/renal congestion. Right coronary blood flow can be augmented in individuals without coronary obstruction with the use of the pure α_1_ agonist, phenylephrine, or with the combined α_1_ and β_1_ agonist, norepinephrine. The improvement in coronary perfusion afforded by these agents must be balanced against the potential negative consequences of vasopressor-induced elevations in pulmonary afterload and myocardial oxygen demands.

Chronic PAH patients with risk factors for coronary disease should rapidly undergo diagnostic evaluation with measures quickly instituted in those subjects with obstructive lesions to prevent myocardial necrosis and irreversible myocardial failure. RV infarction secondary to a coronary stenosis typically occurs with a proximal occlusion of the right coronary artery but can also be seen with left anterior descending (moderator branch artery) and circumflex artery occlusions. The EKG will most commonly show signs of an inferior or inferoposterior myocardial infarction with >1mm of ST elevation in leads II, III, and aVF with or without concomitant ST changes in lead V1. A right-sided EKG with ST elevation in V4R has a sensitivity of 88% and specificity of 78% for diagnosing RV infarction [10]. Anti-platelet and antithrombin therapy with prompt percutaneous coronary revascularization is often necessary. Such patients are typically poor operative candidates.

#### Sepsis

Patients with chronic PAH are at risk for the same infections encountered by the general public. However, due to their high frequency of hospital exposure and routine utilization of indwelling central venous catheters for administration of vasoactive therapies, patients with chronic PAH are also at an increased risk for bacteremia (often with multidrug resistant organisms). In individuals with chronic PAH, cardiodepressive substances present in a septic milieu can precipitate acute RV failure. With superimposed peripheral vasodilatation, systemic pressures can drop below pulmonary pressures and Eisenmenger-type physiology can result with a potential for cyanosis in those patients with a patent foramen ovale.

Management of RV failure in chronic PAH patients with septicemia should be aimed at restoring systemic vascular resistance and treating the underlying pathogen(s). Intravascular volume management in septic patients with hypotension and concomitant RV failure is difficult. LV preload is dependant upon pulmonary venous return, but this is often impaired in PAH due to RV systolic dysfunction. However, aggressive fluid resuscitation for blood pressure management during sepsis should be avoided in PAH patients. Instead, measures should be undertaken to improve systemic pressures and end-organ perfusion with vasopressor and/or inotropic therapies. Dobutamine, dopamine, or norepinephrine can be initiated, but the tachycardia induced by these agents may be poorly tolerated. Phenylephrine should be employed cautiously, as the potent α_1_ agonist properties affect both systemic and pulmonary vasculatures without providing inotrope support. Combination therapy may be required to achieve hemodynamic stability.

Once renal perfusion is improved, diuresis should be attempted in those individuals who develop volume overload during resuscitation measures. Due to splanchnic congestion and diuretic resistance in hypertrophied renal tubules, high-dose bolus diuretic therapy (with or without metolazone or a hydrodiuril primer) or continuous diuretic infusions may be necessary. A reduction in RV volumes will improve the Frank-Starling relationship and subsequently increase RV cardiac output. 

#### Pulmonary Embolism

Patients with chronic pulmonary hypertension are at risk for pulmonary embolism, both from *in situ* pulmonary arterial clot formation and central venous catheter and deep venous sources. Recommendations on pulmonary embolism management will be provided later in this review.

#### Arrhythmias

Patients with chronic PAH are at risk for atrial and ventricular tachyarrhythmias, both of which are poorly tolerated and can lead to rapid RV failure and clinical decompensation. Tricuspid regurgitation is common in PAH, the severity of which increases as RV failure and annular dilatation progress. As the right atrium dilates to accommodate the regurgitant volume, fibrosis ensues. Areas of macro or microreentry can subsequently develop and the nidus for atrial flutter and fibrillation, respectively, is created. Periods of increased atrial stretch (occurring with volume overload) and premature atrial contractions are enough to trigger reentry in those patients who are susceptible. Since many individuals with PAH are young and have fast AV nodal conduction velocities, they are capable of generating very rapid ventricular chronotropic responses. In the setting of PAH with a dysfunctional RV and preload dependant LV, a shortened diastolic LV filling period can be catastrophic.

Therapy for atrial fibrillation and flutter should focus on rapid rhythm or rate control. Hemodynamically unstable patients should promptly undergo synchronized direct current cardioversion (DCCV). In patients without significant hemodynamic embarrassment, rhythm control can be attempted with amiodorone therapy. Beta-blockers and calcium channel blockers should be used with extreme caution due to their negative inotropic properties. Digoxin loading can be attempted for rate control, but patients with renal dysfunction and those on epoprostenol therapy may require dose adjustments with close monitoring of serum digoxin levels. 

Metabolic disturbances, including hypoxia and acidosis, often develop as RV failure progresses in end-stage PAH and such disturbances become a source for atrial and ventricular arrhythmias that arise from myocardial automaticity. Atrial tachycardia and multifocal atrial tachycardia are two such supraventricular rhythms commonly encountered in RV failure. Both respond poorly to DCCV and therapy should be directed at correcting the underlying cause with rate control as discussed above. Automatic ventricular tachycardias are an ominous sign for patients with chronic PAH and RV failure. Unstable patients should undergo immediate defibrillation and efforts to correct metabolic insults (hyperkalemia, acidosis, hypoxia) should be undertaken. Recurrent arrhythmias can be temporarily quiesced with antiarrhythmics such as amiodorone.

#### Iatrogenic Causes of Right Ventricular Failure

Patients with severe chronic PAH poorly tolerate even small changes in hemodynamics. Right ventricular failure can result suddenly if vasoactive medications are acutely withdrawn or inappropriately initiated. Epoprostenol therapy has a half-life of about 2-5 minutes and severe, and potentially fatal, rebound pulmonary hypertension has been known to occur with acute discontinuation of chronic epoprostenol infusion. This can also occur with abrupt discontinuation of high dose inhaled nitric oxide. Medications provided for other ailments can also have unfavorable hemodynamic effects on PAH patients. The negative inotropic properties of beta-blockers and some calcium channel blockers can provoke RV decompensation. In the peri-procedure period, sedatives should be used with caution, beginning with small doses to avoid systemic hypotension. Vasodilators, like nitroprusside and milrinone, should be avoided in severe PAH due to the potential for hemodynamic compromise and Eisenmenger-type systemic flow reversals. Finally, in patients requiring mechanical ventilation, high plateau and high positive-end expiratory pressures (PEEP) increase ventricular end-diastolic pressures and can have negative effects on cardiac output.

### Diagnosis of Right Ventricular Failure in Chronic Pulmonary Hypertension

Identification of acute RV failure in chronic PAH requires a compilation of data obtained from the vitals, the physical exam, laboratories, echocardiography, the EKG, and in some instances, Swan-Ganz catheterization. Cardiac physical exam findings common in RV failure include an RV heave, increased pulmonary component of the second heart sound, a right-sided S3, a tricuspid regurgitation murmur, jugular venous distention, and peripheral cyanosis and edema. Hepatomegly and ascites may also be present. Invasive blood pressure monitoring with blood gas measurement is often warranted to guide management of hypotension and hypoxia, respectively. Laboratories may reveal evidence of end-organ hypoperfusion with elevated creatinine and lactate and reduced serum sodium and bicarbonate. Brain natiuretic peptide and troponins levels are frequently elevated due to myocardial stretch and ischemia, respectively. Hepatic transaminase and bilirubin elevations indicate hepatic ischemia and/or congestion. Echocardiography and Swan-Ganz catheterization can also be useful in diagnosing RV failure and will be discussed in detail later in the review.

### Pharmacologic Therapy Specific to Right Ventricular Failure in Chronic Pulmonary Arterial Hypertension:

Any of the above factors can cause an acute worsening of chronic PAH, leading to rapid clinical decompensation and RV failure. While identifying and managing the instigating factor is mandatory, therapy should also be aimed at improving RV failure directly. The judicious management of intravascular volume to reduce RV wall stress and the use of inotropes and pressors to improve cardiac contractility and coronary perfusion, respectively, has already been addressed. In addition, pharmacologic management with inhaled nitric oxide and epoprostenol can be employed.

#### Inhaled Nitric Oxide

Endogenous nitric oxide was originally identified as “endothelium derived relaxation factor” and functions a vasodilator. Inhaled nitric oxide (iNO), a selective pulmonary vasodilator, traverses the endothelium in the precapillary pulmonary arteries and activates a heme moiety on guanate cyclase, inducing smooth muscle relaxation [11]. The half-life of iNO is on the order of seconds and its rapid elimination by hemoglobin limits its vasodilatory properties to the pulmonary vasculature, making it ideal in the setting of systemic hypotension due to RV failure from decompensated PAH.

Inhaled nitric oxide is not approved by the Food and Drug Administration (FDA) for the management of PAH. However, anecdotal data for the use of iNO exists and it is employed in some centers for PAH therapy. Inhaled nitric oxide can be administered *via* face mask or endotracheal tube, starting at 5 ppm and titrating to a maximum of 40 ppm to goal cardiac index. Methemoglobin levels should be monitored every 6 hours and maintained at <5% hemoglobin to avoid methemoglobinemia. With reductions in pulmonary vasoconstriction, LV preload will improve and cardiac index will increase. Error should not be made in titrating iNO to pulmonary arterial pressures, as an increase in RV stroke volume with vasodilatation may result in increased pulmonary artery pressures. Care should also be taken to avoid abrupt discontinuation of iNO, weaning in 5 ppm decrements until a dose of 5 ppm is reached. 

#### Epoprostenol

Prostaglandin I_2_ (PGI_2_) is endogenously produced by endothelial cells and serves as a potent systemic and pulmonary vasodilator with anti-proliferative and anti-thrombotic properties [12]. It functions in opposition to thromboxane A_2_ which induces vasoconstriction and platelet aggregation [13]. In the presence of hypoxemia, the response of smooth muscle cells to PGI_2_ is reduced. In patients with PAH, thromboxane levels are increased relative to PGI_2_ and this imbalance increases with progressive hypoxemia and RV dysfunction. 

Given its vasoreactive properties, PGI_2_ has been synthetically reproduced in its salt form, epoprostenol, for management of PAH. Epoprostenol’s systemic half-life is short (2-5 minutes), limiting therapy to continuous intravenous infusion. In addition to its vasodilatory properties, epoprostenol has been shown to stimulate the release of NO, to improve RV function (possibly through increased inotropy), and to increase the clearance of the endogenous vasoconstrictor endothelin [3]. Studies have shown improved quality-of-life, NYHA functional class, and reduced mortality with epoprostenol therapy in chronic PAH [14]. While no studies to date have been performed in patients with acute RV failure, its use in the intensive care unit should not be prohibited. Many patients with chronic PAH are already on epoprostenol therapy, and vasodilator infusion should be continued or even up-titrated. Abrupt discontinuation of intravenous epoprostenol should be avoided due to the possibility of rebound pulmonary hypertension. 

In PAH patients naïve to epoprostenol, therapy should ideally be begun when the patient is hemodynamically stable (systolic blood pressures >80 mmHg). Dosing protocols vary from center to center. In the acute setting, a continuous infusion is often started at 2 ng/kg/min and titrated by 0.5–1.0 ng/kg/min every 30 minutes (maximum dose 12 ng/kg/min) until the desired cardiac index is achieved or side-effects (nausea, headache, diarrhea, myalgias) are encountered. Epoprostenol can be used in concert with iNO, and may assist in weaning iNO and vasopressor therapies in favor of long term therapy with epoprostenol.

#### Endothelin Antagonists

Endothelin-1 is produced mostly by endothelial cells and has autocrine and paracrine properties, stimulating endothelial smooth muscle cell vasoconstriction, platelet aggregation, extracellular matrix synthesis, and cytokine expression. In patients with chronic PAH, levels of endothelin-1 are elevated and increase proportionally to the degree of pulmonary oxygen desaturation and pulmonary vascular hypertension [15,16]. Bosentan and ambrisentan are orally active endothelin antagonists that inhibit the endothelin system by blocking endothelin receptors, including those in the pulmonary vasculature. While placebo-controlled trials with endothelin antagonists in chronic PAH have demonstrated improved six minute walk distance, dyspnea, and cardiac index, there is currently no data for use of these agents in the setting of acute RV failure [17,18].

### Atrial Septostomy

**Fig. (4):**
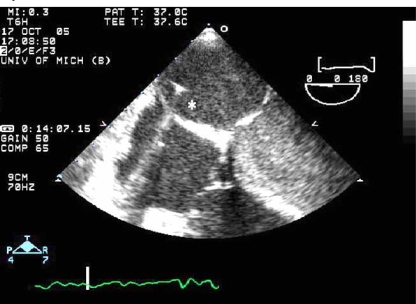
Atrial septostomy. An image of an atrial septal puncture is show above using transesophageal echocardiography during the procedure. Using a specialized catheter and fluoroscopy, an atrial septal puncture is made in the region of the fossa ovalis and balloon dilation is performed. The catheter (^*^asterix) can be seen traversing the septum from the right atrium into the left atrium.

In selected chronic PAH patients with severe RV failure, atrial septostomy may allow for hemodynamic stabilization by reducing RV preload. The concept arose from observations that patients with chronic PAH who had patent foramen ovales (~ 25% of the population) lived longer than those who did not [19,20]. The procedure takes place in the interventional cardiology suite and involves creating an interatrial connection by means of an atrial septal puncture with subsequent balloon dilation (Fig. **4**). In patients with elevated right-sided pressures, blood flows down a pressure gradient to the left atrium, reducing right-sided wall stress and improving RV cardiac output. While the shunted blood to the left atrium is deoxygenated, systemic oxygen transport may improve due to increased left ventricular stroke volumes. Atrial septostomy has been shown to improve clinical symptoms and hemodynamic parameters in hemodynamically stable chronic PAH patients with preserved RV function who are refractory to medical therapy [20]. However, studies suggest the procedure may be of less benefit, and may even be dangerous, in the setting of acute RV failure due to reduced systemic arterial oxygen saturations and elevations in left atrial pressure that may promote RV ischemia and pulmonary edema, respectively [21].

## RIGHT VENTRICULAR FAILURE IN ACUTE PULMONARY EMBOLISM

Acute pulmonary embolism (PE) is a disorder that affects at least one in every thousand individuals in the U.S., and as many as two-thirds of clinically significant PEs are undiagnosed pre-mortem due to its nonspecific clinical presentation and a poor public awareness of the disorder [22-24]. Risk factors for PE include obesity, immobilization, cigarette use, cancer, surgery, trauma, pregnancy, oral contraceptives or hormone replacement therapies, and a prior history of PE or known hypercoagulable disorder. The clinical presentation of PE varies from an asymptomatic small pulmonary embolus with low mortality to a massive PE resulting in RV failure, shock, and/or death. The pathophysiology, diagnosis, management, and prognosis of acute RV failure in the setting of PE will be discussed below.

### Pathophysiology of Right Ventricular Failure in Acute Pulmonary Embolism

The hemodynamic response to an acute PE depends not only the size of the embolus and degree of pulmonary vasculature obstruction, but also on the physiologic reaction to the vasoreactive substances released in response to the event and the cardiopulmonary status of the patient at baseline. In individuals without pre-existing cardiopulmonary disease, 25-30% of the pulmonary vasculature must be occluded before pulmonary pressures begin to rise [25]. Likewise, the normal RV can generate up to a mean pulmonary artery pressure of 40 mmHg acutely, requiring 50-75% of the pulmonary vascular to be obstructed by clot before RV failure ensues [25,26]. Hypoxia induced by the emboli causes localized vasoconstriction. In addition, platelet and thrombin-rich clots stimulate the release of vasoactive mediators, such as serotonin, thromboxane, and histamine, which lead to a further increase in pulmonary vascular resistance. While a patient may initially appear clinically stable, hypotension and shock can rapidly evolve as the RV begins to fail under the high afterload conditions.

While the mechanisms of acute RV failure in PE are similar to those seen in patients with acute RV failure in the setting of chronic PAH, patients without causes for preexisting pulmonary hypertension lack the compensatory right ventricular hypertrophy meant to reduce wall stress in a high afterload state. The presence of a pre-existing coronary occlusion or other cardiopulmonary comorbidities will also result in hemodynamic compromise at a lower level of pulmonary arterial obstruction.

### Prognosis of Patients with Pulmonary Embolism and Right Ventricular Failure

The prognosis of PE correlates most directly with the degree of symptomatic hemodynamic compromise and asymptomatic RV dysfunction. While cardiogenic shock occurs in less than 5% of patients with PE, the mortality of patients with cardiogenic shock ranges from 25-40%, and is as high as 65-95% in patients requiring cardiopulmonary resuscitation [27-32]. The majority of patients who sustain massive PEs (defined as >50% occlusion of the pulmonary vasculature) do not have clinical cardiogenic shock and, therefore, have a much lower mortality than those patients with shock [28-33]. Anatomically massive PEs account for only 50% of the mortality in pulmonary embolism with the remaining fatalities occurring in patients with submassive or recurrent pulmonary emboli. 

In patients who are clinically stable, it appears that RV function is an important prognostic indicator. RV dysfunction is diagnosed on transthoracic echocardiography in up to 30-70% of normotensive PE patients [34-36]. The presence of RV dysfunction without hemodynamic compromise affords a two-ten fold higher risk of short term death compared with normotensive patients without RV dysfunction [34-36]. The development of RV failure can be delayed by 12-48 hours as the cycle of elevated afterload, RV dilation, tricuspid insufficiency, and RV hypokinesis unfold. Recurrent pulmonary emboli can also occur secondary to embolization of persistent deep venous clot, leading to rapid clinical decompensation. Thus, heightened suspicion for the presence of RV failure or RV dysfunction should be had for every patient admitted with acute pulmonary embolism, regardless of initial hemodynamic stability. 

### Diagnosis of Acute Right Ventricular Failure in Pulmonary Embolism

Rapid diagnosis of RV failure in patients with PE is imperative. Patients with fulminant RV failure in the setting of acute PE usually present dramatically with the typical symptoms of PE along with syncope, chest pain, cardiogenic shock, hypoxia, and/or cardiac arrest. The physical exam is similar to that encountered in chronic PAH patients with acute RV failure. The jugular venous pulse is often elevated with a prominent *v* wave. A parasternal RV heave may be palpable. On auscultation, a tricuspid murmur, increased S2, and in ~25% of patients, a right-sided S4 may be present [37]. In patients with normal LV function at baseline, the point of maximum impulse is hyperdynamic and a systolic flow murmur may be audible.

#### EKG Abnormalities

There are several EKG abnormalities associated with PE, but none are highly specific or sensitive. In patients with RV failure, the EKG may show sinus tachycardia, signs of RV strain, repolarization abnormalities, or ischemia including a complete or incomplete right bundle branch block, a right ward axis (>90 degrees), an S1Q3T3 pattern, a Qr in lead V1, ST elevation in V1, or precordial T-wave inversions. However, approximately two-thirds of patients with massive or submassive PEs exhibit no such changes on EKG [38]. Two studies have demonstrated that T-wave inversions in the precordial leads correlate with both the severity of the PE and the presence of RV dysfunction [39,40]. Normalization of T-wave abnormalities implies a favorable outcome [39,40]. In a blinded study of 75 patients with PE, the presence of a Q wave ≥0.2 mV in lead V1 in patients with a QRS <120 ms had a specificity of 97% and a sensitivity of 31% for predicting moderate to severe RV dysfunction [41]. An increased mortality has also been found in acute PE patients presenting with atrial fibrillation, a low voltage QRS, and premature ventricular contractions, again likely reflecting the degree of RV failure and hemodynamic compromise [42]. While the EKG can not be used as a diagnostic tool for PE, it may suggest the presence of underlying RV dysfunction and/or reveal important alternative diagnoses (such as myocardial infarction) in patients presenting with hypotension.

#### Laboratory Abnormalities

It is not uncommon for patients with acute PE to present with elevations in cardiac troponins due to subendocardial ischemia in the setting of decreased coronary perfusion and increased myocardial demand. Cardiac troponins are elevated in 7-32% of patients with PE and are strongly correlated with the presence of RV dysfunction on echocardiography [43-45]. Patients presenting with PE and positive troponins have an odds ratio for death of 15.2-21.0 and troponins remain a prognostic tool independent of echocardiography, patient age, or degree of hypoxemia [43,46]. Likewise, plasma brain natriuretic peptide (BNP) levels have also been identified as a predictor of adverse outcome, likely reflecting the degree of myocardial stretch or pressure increase encountered by the afterloaded RV [47]. While elevations of BNP are correlated with increased mortality in acute PE, a low BNP level (50-90 pg/ml) does not guarantee an uncomplicated hospital course [48].

#### Transthoracic Echocardiography

Transthoracic echocardiography (TTE) is very useful in guiding the management of patients with suspected RV failure in the setting of both acute PE and chronic PAH. Echocardiography can be used to elucidate the severity of pulmonary hypertension and RV dysfunction, may visualize a PE “in transit,” and can identify alternative causes for hypotension (such as LV failure and pericardial effusion). In patients with acute PE, the presence of RV dysfunction affords an increased mortality and a 14% risk for recurrent pulmonary embolism [30, 49].

Abnormalities of the RV noted on TTE in the setting of acute massive PE and chronic PAH include RV hypokinesis, RV pressure and volume overload, and LV diastolic abnormalities incurred from a failing RV. A pulmonary vascular obstruction of >30% has been shown to correlate with the presence of RV dysfunction on echo [50]. In a prospective study of 209 patients with documented PE, TTE was found to have a 100% negative predictive value for predicting PE related death on the basis of RV dysfunction, but a positive predictive value of only 5% [36]. Thus, transthoracic echo is useful for early detection of RV dysfunction in normotensive patients who are at higher risk for hemodynamic decompensation, but with a low specificity (61%). 

Signs of RV dysfunction with dilation on TTE include: 1) a ratio of RV to LV end diastolic diameter >1 in the apical four chamber view, or 2) an RV end diastolic diameter >30 mm and/or loss of inspiratory collapse of the inferior vena cava (IVC). In contrast to the global hypokinesis seen in PAH patients with RV failure, PE patients may exhibit sparing of the RV apex with hypokinesis of the RV free wall and base –a finding termed the “McConnell sign.” The McConnell sign has been shown in one study to have a specificity of 94% and sensitivity of 77% for diagnosing PE [51].

When the RV becomes pressure overloaded, the interventricular septum deviates toward the LV, creating a “D” shaped septum on parasternal short axis TTE images. The displaced septum impedes diastolic mitral inflow, increasing the demand on atrial systole for LV diastolic filling. This results in a diastolic filling pattern on Doppler interrogation of the transmitral inflow called “E to A reversal,” with an increased A (atrial systole) wave compared to the reduced E (passive atrial filling) wave. 

An estimation of pulmonary artery systolic pressure can be obtained from the regurgitant jet of the tricuspid valve. The pulmonary artery systolic pressure (PAP) is estimated from the right ventricular systolic pressure (RVSP), according to the formula: PAP = RVSP + estimated right atrial pressure. The RVSP is obtained from the velocity (v) of tricuspid regurgitant jet, such that RVSP = 4v^2^. Right atrial pressure is estimated from atrial size or IVC engorgement. While easy to obtain, the RVSP can be greatly underestimated or unobtainable in the setting of an inadequate Doppler signal (due to obesity, pulmonary hyperinflation, mechanical ventilation, or angulation of the heart) or with poor alignment of the transducer probe with the tricuspid jet. In patients with pulmonary embolism, absence of any tricuspid regurgitation on a quality echo makes the presence of severe pulmonary hypertension unlikely, while a tricuspid jet velocity > 3.7 m/s or an RV wall thickness > 5 mm suggests the presence of preexisting cardiopulmonary disease, such as chronic PAH [52].

**Fig. (5):**
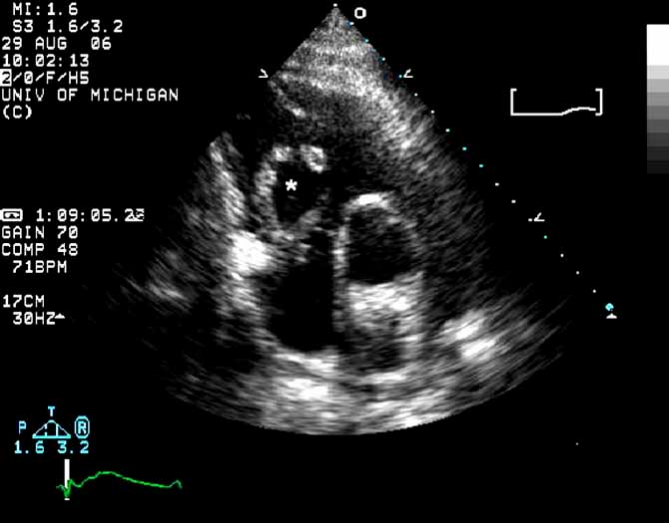
Pulmonary embolism in transit. This short axis tranthoracic echocardiogram image shows an embolism (^*^asterix) passing through the tricuspid valve into the right ventricle.

Rarely, TTE or transesophageal echocardiography (TEE) can be diagnostic of PE, visualizing the presence of clot, termed a PE “in transit,” in the IVC, RV, or pulmonary artery (Fig. **5**). TEE can be employed at the bedside in patients too unstable to undergo radiologic imaging. In experienced centers, the sensitivity of TEE for detecting central PE is 80-97% with a specificity of 84-100% [53,54]. Beyond the proximal pulmonary arteries, however, the sensitivity of TEE for identifying emboli drops markedly. While TTE and TEE can not rule out the diagnosis of PE, the presence of emboli “in transit” is a clear indication for therapy. 

#### Swan-Ganz Catheterization

Pulmonary artery Swan-Ganz catheterization can be useful in selected patients with chronic PAH or PE and hypotension of unclear etiology, allowing one to differentiate RV failure from other causes of hypotension, including sepsis and hypovolemia. Echocardiography is useful prior to catheterization to screen for RV clot that may complicate catheter placement. 

The results of the Swan must be taken in context of pre-existing cardiopulmonary disease. In patients with pulmonary emboli who do not have pre-existing cardiopulmonary disease, an elevation in the right atrial (RA) pressure suggests the presence of a high pulmonary vascular clot burden. When the RA pressure is ≥10 mmHg in a previously normal RV, there is likely ≥50% obstruction to the pulmonary vasculature [25]. While patients with small PEs typically have normal or supranormal cardiac output with normal or mildly elevated RA pressures, patients with chronic PAH and other causes for cardiopulmonary dysfunction will have a reduction in cardiac output and mixed venous oxygen saturation with a greater increase in RA pressure. As RV failure progresses, RA pressures elevate while pulmonary pressures often decline to near normal values due to reductions in RV stroke volume. The presence of a reduced cardiac output with a normal RA pressure suggests the presence of alternative etiologies for hypotension.

In chronic PAH patients with PE, RA pressures on catheterization do not correlate reliably with the severity of vascular obstruction. In complicated hypotensive patients with PE and an unclear preexisting cardiopulmonary disease history, it has been suggested that a mean pulmonary artery pressure to percent of angiographic obstruction ratio ≥1.0 represents a vascular hypertensive response in a patient who has a concomitant limitation in cardiac reserve [26].

### Management of Right Ventricular Failure in Acute Pulmonary Embolism

Management of patients with acute PE is dictated by the presence or absence of hemodynamic compromise. Patients presenting with PE in cardiogenic shock should immediately receive vasopressor support, anticoagulation, and then be considered for thrombolysis and/or embolectomy where available. While heparin reduces the morbidity and mortality from recurrent PEs, it does not affect the clot(s) causing the initial hemodynamic insult. Thrombolytic agents accelerate clot lysis by converting plasminogen to its active form, plasmin, dissolving intrapulmonary clot and clot that is at risk for subsequent embolization from the deep veins. At present, there are three agents (streptokinase, urokinase, tissue plaminogen activator) approved by the FDA for thrombolysis of pulmonary emboli. All are administered intravenously or angiographically for direct clot lysis in patients without contraindications.

While no large individual trial has demonstrated a statistically significant mortality benefit from lytic therapy compared with heparinization alone in patients with PE, lysis does lead to a more rapid reduction in angiographic clot burden and pulmonary hypertension, restores RV function, and reduces the risk of recurrent pulmonary emboli [55]. However, lytic therapy must be weighed against the 9-22% risk of major bleeding, including a 1-5% risk of intracranial hemorrhage [29,55-58]. 

Metaanalyses of lytic therapy trials have shown that the majority of benefit from thrombolytic therapy is limited to PE patients with hemodynamic instability [35]. While normotensive patients with evidence of RV dysfunction have increased mortality and thrombolytic therapy has been shown to improve RV dysfunction, definitive data demonstrating a significant morbidity and/or mortality benefit from lytic therapy in this population is lacking. Several studies have examined the efficacy of lytic therapy in normotensive patients with RV dysfunction and PE, but they lack uniform tools (TTE *vs*. EKG) and uniform terminology for defining RV dysfunction and/or fail to state how outcome criteria for PE related mortality was assessed [35], fail to confirm the diagnosis of PE [34], or have potential patient selection biases [58]. Despite the high specificity of TTE, the positive predictive value of the modality in predicting in hospital mortality is low (4-5%), potentially subjecting an unnecessarily large number of patients to lytic therapy [35]. Further, studies that limit data to normotensive patients with PE-related RV dysfunction reveal lower short-term mortality differences (4-5%) than trials examining mortality differences (4-14%) in all PE patients with RV dysfunction [35-36]. Thus, further studies are needed to determine which hemodynamically stable patients will benefit from lytic therapy in the setting of RV dysfunction. 

Unstable patients with pulmonary emboli who have failed maximal medical management with pressor resistant hypotension or cardiac arrest, or who have contraindications to thrombolytic therapy can be considered for percutaneous or surgical embolectomy. Catheter based embolectomies include clot aspiration, clot fragmentation, and rheolytic thrombectomy. The percutaneous techniques, however, are limited by the size of clot that can be removed through a small catheter lumen. Surgical embolectomy is not widely available but offers the ability to extract larger clots while providing cardiopulmonary bypass support. The current data available on embolectomy is from case reports or trials with small numbers of patients. But, mortality varies from 16-46%, with the higher mortality found in older studies and in studies that included patients with cardiac arrest [52, 59,60]. Antecedent lytic therapy has not been cited as a contraindication to surgical embolectomy, but an increase in complications should be anticipated. 

Finally, in patients presenting with RV failure, a differentiation between acute and chronic pulmonary embolism is required as management strategies, follow-up, and prognosis differ. Patients with acute PE should have close follow-up after discharge to ensure that chronic thromboembolic pulmonary hypertension does not develop. Treated patients with dyspnea that persists in the outpatient setting should provoke concern for the development of chronic pulmonary embolic hypertension, and radiologic imaging (contrasted computed tomography or ventilation perfusion scanning) should be employed. These patients should be referred to a PAH specialist for consideration of thromboendarterectomy, vasodilator therapy, or lung transplantation as appropriate.

## CONCLUSION

Acute RV failure in the setting of chronic pulmonary hypertension and acute PE portend a high morbidity and mortality. Management strategies should focus on identifying precipitating factors, restoring oxygenation and hemodynamic stability, and employing pharmacologic therapies and/or procedures shown to improve outcome. Consultation with a pulmonary hypertension specialist should be strongly considered to assist with medical management and to guide referral for surgical intervention.
